# Candida glabrata Transcription Factor Rpn4 Mediates Fluconazole Resistance through Regulation of Ergosterol Biosynthesis and Plasma Membrane Permeability

**DOI:** 10.1128/AAC.00554-20

**Published:** 2020-08-20

**Authors:** Pedro Pais, Raquel Califórnia, Mónica Galocha, Romeu Viana, Mihaela Ola, Mafalda Cavalheiro, Azusa Takahashi-Nakaguchi, Hiroji Chibana, Geraldine Butler, Miguel C. Teixeira

**Affiliations:** a Department of Bioengineering, Instituto Superior Técnico, Universidade de Lisboa, Lisbon, Portugal; b iBB-Institute for Bioengineering and Biosciences, Biological Sciences Research Group, Instituto Superior Técnico, Lisbon, Portugal; c School of Biomedical and Biomolecular Sciences, Conway Institute, University College Dublin, Dublin, Ireland; d Medical Mycology Research Center, Chiba University, Chiba, Japan

**Keywords:** CgRpn4, RNA-seq-based transcriptomics, azole resistance mechanisms, ergosterol, regulatory networks

## Abstract

The ability to acquire azole resistance is an emblematic trait of the fungal pathogen Candida glabrata. Understanding the molecular basis of azole resistance in this pathogen is crucial for designing more suitable therapeutic strategies. This study shows that the C. glabrata transcription factor (TF) CgRpn4 is a determinant of azole drug resistance. RNA sequencing during fluconazole exposure revealed that CgRpn4 regulates the expression of 212 genes, activating 80 genes and repressing, likely in an indirect fashion, 132 genes. Targets comprise several proteasome and ergosterol biosynthesis genes, including *ERG1*, *ERG2*, *ERG3*, and *ERG11*. The localization of CgRpn4 to the nucleus increases upon fluconazole stress. Consistent with a role in ergosterol and plasma membrane homeostasis, CgRpn4 is required for the maintenance of ergosterol levels upon fluconazole stress, which is associated with a role in the upkeep of cell permeability and decreased intracellular fluconazole accumulation. We provide evidence that CgRpn4 directly regulates *ERG11* expression through the TTGCAAA binding motif, reinforcing the relevance of this regulatory network in azole resistance. In summary, CgRpn4 is a new regulator of the ergosterol biosynthesis pathway in C. glabrata, contributing to plasma membrane homeostasis and, thus, decreasing azole drug accumulation.

## Abstract (precis)

The ability to acquire azole resistance is an emblematic trait of the fungal pathogen Candida glabrata. Understanding the molecular basis of azole resistance in this pathogen is crucial for designing more suitable therapeutic strategies. This study shows that the C. glabrata transcription factor (TF) CgRpn4 is a determinant of azole drug resistance. RNA sequencing during fluconazole exposure revealed that CgRpn4 regulates the expression of 212 genes, activating 80 genes and repressing, likely in an indirect fashion, 132 genes.

## INTRODUCTION

Infections by fungal pathogens have become a worrying health concern worldwide. *Candida* spp. represent the most common cause of fungal infections and rank as the 4th leading cause of nosocomial bloodstream infections in the United States (1–3). They are responsible for localized superficial infections in healthy individuals but can cause life-threatening scenarios in immunocompromised patients. The ability to rapidly acquire azole resistance has been associated with the increased prevalence of Candida glabrata relative to the most commonly isolated species, C. albicans (1, 4–9). C. glabrata now ranks as the second or third most prevalent *Candida* species, depending on geographical location. While it is the second most commonly isolated species in the United States and northern Europe (7, 10), it ranks as the third most common species in southern Europe and Asia (7, 10, 11) and is less prevalent in South America (10).

Due to their safety profile and availability in both oral and intravenous formulations, azoles have been the most widely used antifungals for decades, both as a first-line treatment and as prophylaxis, especially fluconazole (12). Azoles disrupt ergosterol biosynthesis, the main sterol in fungal plasma membranes, which leads to the loss of membrane structural properties. They act by binding and inhibiting the lanosterol 14-α-demethylase, a key enzyme of the ergosterol biosynthetic pathway encoded by *ERG11* (13, 14). The result is the accumulation of 14-α-methylated sterols, the depletion of ergosterol, and the production of the toxic sterol dimethylcholesta-8,24(28)-dien-3β,6α-diol (DMCDD) in the membrane (15, 16).

Investigation of the transcriptional regulation of antifungal resistance in C. glabrata is focused mostly on the role of the transcription factor (TF) CgPdr1, regarded as a master regulator of clinical acquisition of resistance to azole antifungals by activating the expression of multidrug resistance transporters (17–22). In C. albicans, CaTac1 plays a similar role by regulating the expression of multidrug resistance transporters (23–25). However, the level of sequence similarity between CaTac1 and CgPdr1 is low. Apart from these pathways, knowledge about regulatory networks involved in the response to antifungal stress is scarce, especially for C. glabrata (26).

In this study, we identified the transcription factor Rpn4 as a regulator of fluconazole resistance in C. glabrata. In Saccharomyces cerevisiae, the Rpn4 homolog regulates the expression of proteasome genes, affecting proteasome activity and ubiquitin-mediated proteolysis (27–30), and contributes to the unfolded protein response (UPR) (31). Rpn4 has also been identified as a member of the multidrug resistance network in S. cerevisiae (32, 33). Consistently, the deletion of *ScRPN4* results in increased sensitivity to fluconazole (34, 35) and clotrimazole (36). Moreover, the expression of *ScRPN4* and, consequently, the ubiquitin-proteasome system is regulated by the multidrug resistance regulators Pdr1 and Pdr3 (29). In C. albicans, *RPN4* is also a regulator of proteasome genes, and its absence results in enhanced sensitivity to fluconazole (37). To the best of our knowledge, despite the apparent role of Rpn4 in azole resistance phenotypes, its actual role and mechanistic insights into antifungal resistance have not been addressed.

Given the identification of *CgRPN4* as a new determinant of azole drug resistance in C. glabrata, its role in the transcriptome-wide response to fluconazole was assessed. The determination of Rpn4 target genes enabled the identification of a new regulatory pathway toward azole resistance, which may provide the molecular basis of Rpn4-mediated azole resistance in pathogenic and nonpathogenic yeasts.

## RESULTS

### The transcription factor *CgRPN4* is a determinant of fluconazole resistance in Candida glabrata.

**FIG 1:**
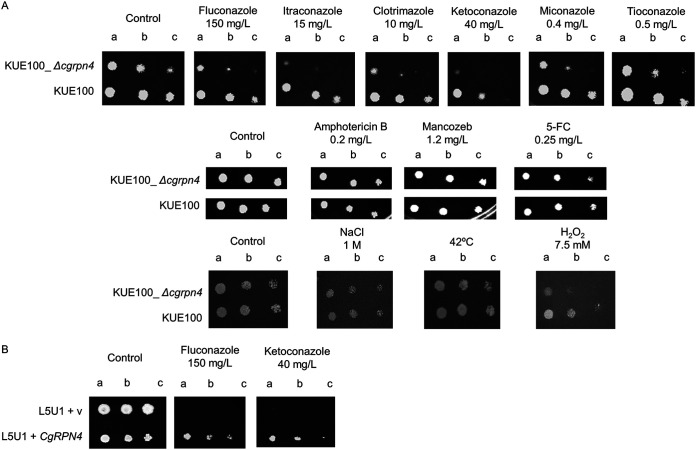
*CgRPN4* confers resistance to fluconazole in C. glabrata. (A) Comparison of the susceptibilities to antifungals and other stress agents, at the indicated concentrations, of the KUE100 and KUE100_Δ*cgrpn4* C. glabrata strains, in BM agar plates, by spot assays. (B) Comparison of the susceptibilities to azole antifungals, at the indicated concentrations, of the L5U1 C. glabrata strain harboring the pGREG576 cloning vector (v) or pGREG576_MTI_*CgRPN4* in BM agar plates supplemented with leucine and 50 μM CuSO_4_ by spot assays. The inocula were prepared as described in Materials and Methods. The cell suspensions used to prepare the spots were 1:5 (lane b) and 1:25 (lane c) dilutions of the cell suspensions used in lane a. The displayed images are representative of results from at least three independent experiments.

Based on the previous identification of Rpn4 as a member of the multidrug resistance network in several yeast species, the role of the uncharacterized *CgRPN4* gene in azole resistance was determined. Figure 1A shows that deleting *CgRPN4* increases sensitivity to 6 different azole drugs compared to the KUE100 wild-type strain. Interestingly, the overexpression of *CgRPN4* in the L5U1 wild-type C. glabrata strain increased its tolerance to fluconazole and ketoconazole (Fig. 1B). Besides susceptibility to azole antifungals, phenotypes to antifungals from other families, amphotericin B and flucytosine (5-FC), were tested. Susceptibility to the fungicide mancozeb was also tested. Other than antimycotic agents, susceptibility to temperature, osmotic stress, and oxidative stress was also tested. The deletion of *CgRPN4* was seen to increase susceptibility only to hydrogen peroxide (Fig. 1A). These results indicate that *CgRPN4* plays a primary role in mediating azole resistance.

The MICs of several antifungals were also used to quantitatively assess drug susceptibility variability between the wild-type strain and the derived Δ*cgrpn4* mutant (Table 1). Consistent with previous results, no MIC differences were attained for amphotericin B or 5-FC. The susceptibility profiles against the imidazole ketoconazole and the triazoles fluconazole, itraconazole, and posaconazole were also determined. The deletion of *CgRPN4* led to an 8-fold increase in susceptibility to ketoconazole and posaconazole and a 2-fold increase in susceptibility to itraconazole. The clinical outcome of these data cannot be directly estimated due to the absence of breakpoints for these antifungals; however, the observed shifts in susceptibility support a relevant role of CgRpn4 in multiple-azole resistance. The deletion of the TF also resulted in a 4-fold increase in susceptibility to fluconazole. Both strains are categorized as intermediately susceptible to fluconazole, with CgRpn4 playing a measurable role in the susceptibility profile.

**TABLE 1:** Susceptibility profiles determined by MIC values for the Δ*cgrpn4* mutant strain and the parental strain^*a*^

Drug	MIC (mg/liter) for strain		Category (WT/mutant)
	WT	Δ*cgrpn4*	
Fluconazole	16	4	I/I
Itraconazole	0.5	0.25	NA
Posaconazole	1	0.125	NA
Ketoconazole	0.500	0.0625	NA
Amphotericin B	0.25	0.25	S/S
5-FC	0.5	0.5	NA

a NA, not applicable; I, intermediate. S, susceptible; WT, wild type.

### Transcriptome-wide changes in response to fluconazole stress in C. glabrata.

We used transcriptional profiling to determine the response of the KUE100 wild-type strain to treatment with the previously determined inhibitory concentration of 150 mg/liter fluconazole. Notably, this concentration of fluconazole is higher than that attained by MIC assays, as it was found to be an inhibitory concentration under the conditions used for cell growth to perform the remaining experiments. Resistance measurements can vary according to the experimental conditions that are used (e.g., cell density, agitation, temperature, and medium), and cell density has been correlated with antifungal resistance (38). Control experiments were performed with reduced cell and fluconazole concentrations to validate the same inhibitory effect with drug concentrations more closely related to those found under physiological conditions (see Fig. S1 in the supplemental material). The fact that the remaining experiments were conducted using a much higher cell concentration than the one used in MIC assays might justify the need for a higher concentration of the antifungal. The expression levels of 44 genes were altered in C. glabrata cells following exposure to fluconazole for 1 h (log_2_-fold change of greater than 0.5 or less than −0.5; Benjamini-Hochberg-adjusted *P* value of <0.05) (Table 2; Table S1). The expression levels of 29 (66%) genes were increased, whereas the expression levels of 15 (34%) were decreased. The genes fall into 11 functional groups: drug resistance, sterol metabolism, and intracellular traffic, which are associated exclusively with the upregulated genes; lipid and fatty acid metabolism; nitrogen metabolism; carbon metabolism; heme homeostasis; mitochondrial function; the stress response; the cytoskeleton/cell cycle; and unknown function. As expected, the expression of the multidrug resistance transporter-encoding gene *CDR1*, a well-known biomarker of azole resistance (39–41), was upregulated 1.75-fold in fluconazole-challenged cells. Additionally, 41% of the upregulated genes are related to sterol metabolism, including components of the ergosterol biosynthetic pathway (*ERG1*, *ERG2*, *ERG3*, *ERG4*, *ERG5*, and *ERG6*) and the azole target *ERG11*. The upregulation of *ERG11* in response to azole stress in C. glabrata and other *Candida* spp. has been reported previously (42–45), as has the upregulation of additional *ERG* genes in cases of azole resistance (46, 47). Two more genes with predicted roles in ergosterol biosynthesis, namely, *HES1* and *CYB5*, are also present in this group. The upregulation of the ergosterol pathway is therefore likely a core response to mild fluconazole stress.

**TABLE 2:** Genes differentially expressed in wild-type C. glabrata cells challenged with fluconazole^*a*^

Functional group	C. glabrata gene	Description	Log_2_-fold change	
			WT FLC/WT control	Δ*cgrpn4* FLC/WT FLC
Drug resistance	*CDR1*	Multidrug transporter of the ABC superfamily, involved in resistance to azoles; expression regulated by Pdr1p; increased abundance in azole-resistant strains; expression increased by loss of the mitochondrial genome	0.81	0.42

Sterol metabolism	*ERG1*^*b*^	Squalene epoxidase with role in ergosterol synthesis; involved in growth under conditions of low oxygen tension	0.83	−0.61
	*ERG2*^*b*^	C-8 sterol isomerase	1.10	−0.71
	*ERG3*^*b*^	Delta-5,6-sterol desaturase; C-5 sterol desaturase; predicted transmembrane domain and endoplasmic reticulum binding motif; gene used for molecular typing of C. glabrata strain isolates	1.07	−0.63
	*ERG4*	Putative C-24 sterol reductase	0.83	−0.46
	*ERG5*	Putative C-22 sterol desaturase	0.58	−0.24
	*ERG6*	C-24 sterol methyltransferase; mutation confers resistance to amphotericin B and nystatin and increased sensitivity to azoles	0.52	−0.38
	*ERG11*^*b*^	Putative cytochrome P-450 lanosterol 14-alpha-demethylase; target enzyme of azole antifungal drugs; increased protein abundance in azole-resistant strains	0.96	−0.65
	*ERG24*	Ortholog(s) has delta-14-sterol reductase activity and roles in cellular response to drugs, ergosterol biosynthetic processes, filamentous growth of a population of unicellular organisms in response to a biotic stimulus, and pathogenesis	0.53	−0.32
	*ERG25*	Ortholog(s) has C-4 methylsterol oxidase activity, role in ergosterol biosynthetic process, and endoplasmic reticulum membrane and plasma membrane localizations	1.02	−0.41
	*ERG29*	Ortholog(s) has roles in cellular iron ion homeostasis, ergosterol biosynthetic process, and mitochondrion organization and has endoplasmic reticulum and nuclear envelope localizations	0.84	−0.43
	*HES1*^*b*^	Ortholog(s) has oxysterol binding, sterol transporter activity, and roles in endocytosis, exocytosis, maintenance of cell polarity, piecemeal microautophagy of the nucleus, and sterol transport	1.48	−0.96
	*CYB5*	Ortholog(s) has electron carrier activity, role in ergosterol biosynthetic process, and endoplasmic reticulum membrane localization	0.73	−0.43

Lipid and fatty acid metabolism	*CSR1*^*b*^	Ortholog(s) has phosphatidylinositol transporter activity	0.59	−0.90
	*HBN1*	Ortholog(s) has oxidoreductase activity acting on NAD(P)H, nitrogenous group as acceptor activity, and roles in cellular response to oxidative stress and negative regulation of fatty acid metabolic process	−0.51	−0.10
	*CAGL0A03740g*^*b*^	Ortholog(s) has roles in fatty acid beta-oxidation and long-chain fatty acid catabolic processes and has peroxisome localization	−0.53	1.13

Stress response	*RTA1*^*b*^	Putative protein involved in 7-aminocholesterol resistance; gene is upregulated in azole-resistant strains	0.80	0.51
	*RAD14*^*b*^	Ortholog(s) has damaged DNA binding, zinc ion binding activity, and roles in UV damage excision repair, nucleotide excision repair involved in interstrand cross-link repair, nucleotide excision repair, and DNA damage recognition	0.52	−0.53
	*SSA3*	Heat shock protein of the HSP70 family	−0.55	0.06

Nitrogen metabolism	*PUT1*^*b*^	Ortholog(s) has proline dehydrogenase activity, role in the proline-catabolic process to glutamate, and mitochondrion localization	0.57	0.72
	*MEP2*	Ortholog(s) has high-affinity secondary active ammonium transmembrane transporter activity and methylammonium transmembrane transporter activity	−0.50	−0.04

Carbon metabolism	*PBI1*^*b*^	Has domain(s) with predicted alcohol *O*-acetyltransferase activity and role in alcohol metabolic process	2.11	−1.93
	*ATF2*	Putative alcohol acetyltransferase involved in steroid detoxification; gene is upregulated in azole-resistant strains	0.70	−0.09
	*MLS1*^*b*^	Ortholog(s) has malate synthase activity; roles in acetate catabolic process, carbon utilization, fatty acid catabolic process, and glyoxylate cycle; and cytosol, glyoxysome, and peroxisomal matrix localizations	−0.53	0.74

Heme biosynthesis	*HEM13*^*b*^	Putative coproporphyrinogen III oxidase; protein differentially expressed in azole-resistant strains	0.81	−0.67
	*HEM14*^*b*^	Ortholog(s) has oxygen-dependent protoporphyrinogen oxidase activity, role in heme biosynthetic process; and cytosol, mitochondrial inner membrane, and nucleus localizations	0.51	−0.70
	*HMX1*	Ortholog(s) has heme oxygenase (decyclizing) activity and roles in cellular iron ion homeostasis, heme catabolic process, response to carbon monoxide, and response to oxidative stress	−0.62	0.40

Cytoskeleton/cell cycle	*MSC7*^*b*^	Ortholog(s) has role in reciprocal meiotic recombination and cytosol, endoplasmic reticulum, and nucleus localizations	0.88	−0.56
	*NCE102*	Ortholog(s) has roles in actin cytoskeleton organization, eisosome assembly, establishment of protein localization to the plasma membrane, negative regulation of protein phosphorylation, and more	−0.55	0.43
	*XBP1*	Ortholog(s) has RNA polymerase II transcription factor activity, sequence-specific DNA binding, and sequence-specific DNA binding activity	−0.69	0.47
	*FMP45*	Ortholog(s) has roles in ascospore formation and cellular response to drugs and has fungal-type cell wall organization	−0.69	0.47

Mitochondrial function	*COX26*	Ortholog(s) has mitochondrial respiratory chain complex IV and mitochondrial respiratory chain supercomplex localization	−0.64	0.12

Intracellular traffic	*SRO7*	Ortholog(s) has Rab GTPase binding and SNARE binding activities and roles in Golgi-to-plasma membrane transport, establishment of cell polarity, exocytosis, and small GTPase-mediated signal transduction	0.51	−0.09

Unknown function	*CAGL0M11660g*^*b*^	Has domain(s) with predicted hydrolase activity	0.67	1.81
	*CAGL0G05632g*^*b*^	Ortholog(s) has cytoplasm localization	−0.52	1.32
	*CAGL0K07337g*^*b*^	Has domain(s) with predicted ion channel activity, role in ion transport, and membrane localization	−0.63	0.66
	*CAGL0A02277g*	Protein of unknown function	−0.72	−0.11
	*PET10*	Ortholog(s) has lipid particle localization	−0.51	0.45
	*MUP1*	Protein of unknown function	−0.78	0.47
	*SET4*	Ortholog of S. cerevisiae SET4 and S. cerevisiae S288C YJL105W	1.14	−0.30
	*CAGL0L06776g*	Has domain(s) with predicted sequence-specific DNA binding and transcription factor activities, zinc ion binding activity, and role in regulation of transcription, DNA templated	0.85	0.33
	*CAGL0L08547g*	Protein of unknown function	0.76	−0.05
	*CAGL0J00297g*	Ortholog(s) has endoplasmic reticulum localization	0.63	−0.41
	*CAGL0G00594g*	Ortholog(s) has Golgi apparatus and endoplasmic reticulum localization	0.61	−0.31

a The effect of the CgRpn4 deletion on the expression pattern is also shown. FLC, fluconazole; ABC, ATP-binding cassette.

b Genes whose expression levels are significantly altered in the absence of CgRpn4.

In turn, genes involved in the heme biosynthetic pathway are upregulated (*HEM13* and *HEM14*), while a gene involved in heme degradation is downregulated (*HMX1*). This profile suggests that the increased synthesis of heme, a vital prosthetic group of Erg11, may be required to accompany the increased expression of Erg11, aiming at increased levels of functional Erg11 molecules.

### Role of CgRpn4 in transcriptome-wide changes occurring in response to fluconazole in C. glabrata.

**FIG 2:**
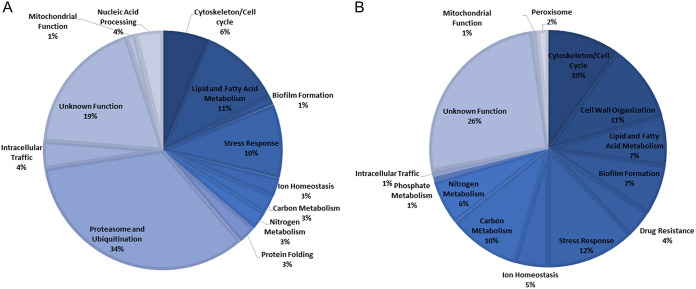
*CgRPN4*-regulated functional groups. Differentially expressed genes in exponential-phase KUE100_Δ*cgrpn4* C. glabrata cells compared to KUE100 cells after 1 h of fluconazole exposure. (A) CgRpn4-activated genes (downregulated in the mutant strain). (B) CgRpn4-repressed genes (upregulated in the mutant strain).

In order to study the role of CgRpn4 in the response of C. glabrata to fluconazole, gene expression changes occurring upon fluconazole exposure in the Δ*cgrpn4* mutant strain were compared to those observed in the wild-type strain. The expressions of 80 genes were found to be activated by CgRpn4, while 132 genes were found to be repressed, possibly in an indirect fashion, upon fluconazole exposure (Table S2). The most prevalent functional groups activated by CgRpn4 include the proteasome and ubiquitination, lipid and fatty acid metabolism, and the stress response (Fig. 2A), whereas repressed genes are enriched in cell wall organization and carbon metabolism (Fig. 2B).

**FIG 3:**
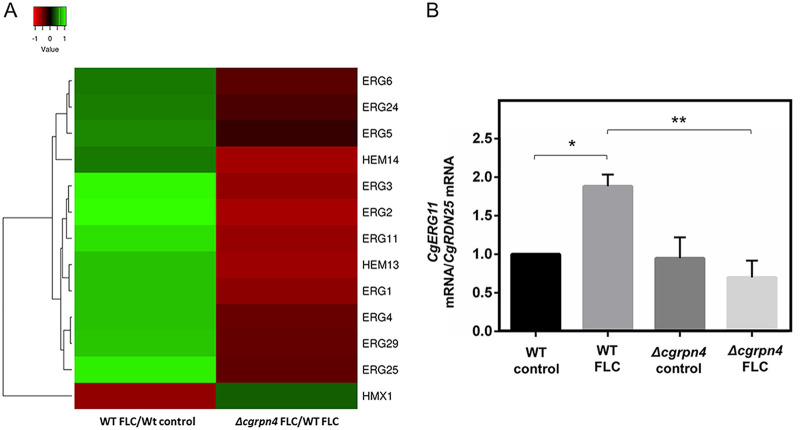
Comparison of ergosterol- and heme-related gene expression patterns in wild-type and Δ*cgrpn4* cells upon fluconazole stress. (A) Expression profiles of differentially expressed *ERG* and *HEM* genes in at least one data set. C. glabrata gene names are shown. In cases where no gene name has been assigned, the designation of its S. cerevisiae homolog is shown. (B) Comparison of the variation of *CgERG11* transcript levels determined by RT-PCR in KUE100 and KUE100_Δ*cgrpn4* cells under control conditions or after 1 h of fluconazole exposure. Transcript levels of *CgRDN25* were used for normalization. Expression values are the averages from at least three independent experiments. Error bars represent the corresponding standard deviations. *, *P* < 0.05; **, *P* < 0.01.

The comparison of this regulon with differentially expressed genes in fluconazole-challenged wild-type cells enabled the identification of 18 genes that respond to fluconazole only when *CgRPN4* is present (Table 2). The most enriched functional group in this set comprises genes from the ergosterol biosynthetic pathway, namely, *ERG1*, *ERG2*, *ERG3*, and *ERG11*. A putative gene involved in the regulation of ergosterol synthesis in S. cerevisiae (*HES1*) (48, 49) is also found in this set. Interestingly, two genes involved in heme biosynthesis are also present: *HEM13* and *HEM14*. As described previously, heme is a key prosthetic group of several ergosterol biosynthesis enzymes, and increased Hem13 protein levels were detected in an azole-resistant strain, concurrently with Erg11 (41). It is important to note that the upregulation of ergosterol biosynthesis is the most dramatic response to fluconazole stress and that CgRpn4 functions as an activator of ergosterol and heme biosynthesis (Fig. 3A). Moreover, the activation of *ERG* and *HEM* genes constitutes a specific role of this TF during fluconazole stress, as CgRpn4 does not regulate their basal expression (Table S3). Altogether, these data indicate that the activation of the ergosterol biosynthetic pathway, mediated by CgRpn4, could be a major regulatory mechanism of azole antifungal resistance. The transcriptome data were validated by real-time PCR (RT-PCR) quantification of *CgERG11* expression in the wild type and the Δ*cgrpn4* mutant. The attained *CgERG11* activation after 1 h of fluconazole exposure is comparable to the expression levels determined by the transcriptomics approach, as was the decreased fold change in the mutant strain (Fig. 3B).

CgRpn4 was found to regulate the expression of several additional genes irrespective of fluconazole treatment. The largest functional group includes proteasome and ubiquitination genes. This group comprises exclusively genes activated by CgRpn4, in accordance with its role in both C. albicans and S. cerevisiae as an activator of proteasome genes (27, 28, 50). The high enrichment of proteasome subunit-encoding genes in the CgRpn4 regulon strongly indicates that its physiological function is conserved with other species.

Genes encoding five multidrug resistance transporters are repressed by CgRpn4: the major facilitator superfamily (MFS) transporters encoded by *CAGL0L10912g*, *CAGL0B02343g*, *TPO1_1*, and *QDR2* as well as the ABC transporter *YOR1*. Of these, *YOR1*, *TPO1_1*, and *QDR2* have been implicated in azole resistance (18, 19, 22). This indicates that CgRpn4 does not activate the expression of drug transporters, reinforcing its role as an activator of ergosterol biosynthesis as the main mechanism of fluconazole resistance.

### CgRpn4 is activated upon fluconazole stress, leading to its nuclear accumulation.

In order to examine possible CgRpn4 activation mechanisms, we investigated its subcellular localization. CgRpn4 was fused to green fluorescent protein (GFP), expressed via the pGREG576_MTI_*CgRPN4* plasmid in C. glabrata cells grown to mid-exponential phase in basal medium (BM) supplemented with 50 μM Cu_2_SO_4_ to induce fusion protein expression, and then transferred to fresh medium (control) or to fresh medium containing 150 mg/liter fluconazole. After 1 h of incubation, cells were inspected by fluorescence microscopy.

**FIG 4:**
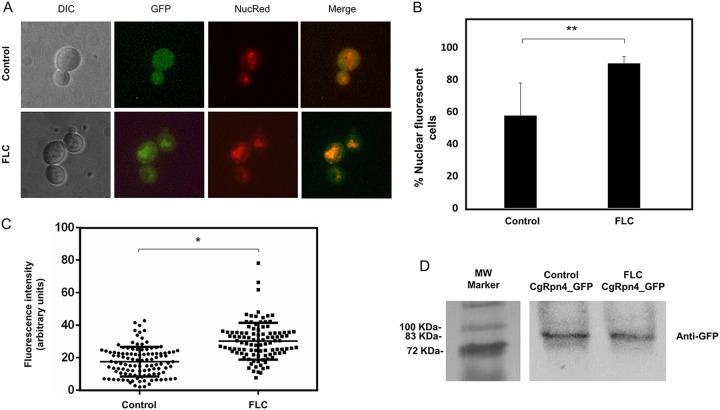
CgRpn4 increases its relative distribution to the nucleus upon fluconazole stress. The subcellular localization of fluorescence in exponential-phase L5U1 C. glabrata cells harboring the pGREG576_MTI_*CgRPN4* plasmid after 5 h of recombinant protein production under control conditions or after 1 h of fluconazole exposure was assessed. (A) Representative images of CgRpn4_GFP localization in L5U1 C. glabrata cells. DIC, differential interference contrast; GFP, green fluorescent protein; NucRed, nuclear stain; merge, GFP-NucRed overlap images. (B) Percentage of cells showing nuclear localization of the CgRpn4_GFP fusion protein under control conditions or after 1 h of fluconazole exposure. Error bars represent the corresponding standard deviations. *, *P* < 0.01. (C) Comparison of nuclear fluorescence intensities of CgRpn4_GFP in L5U1 C. glabrata cells under control conditions or after 1 h of fluconazole exposure. The estimation of nuclear signal intensity was calculated after deduction of the cytoplasm intensity and correction for background intensity. Error bars represent the corresponding standard deviations. *, *P* < 0.05. (D) Western blot detection of the CgRpn4_GFP fusion protein under control conditions or after 1 h of fluconazole exposure. Immunoblotting was carried out using a mouse anti-GFP antibody. The displayed images are representative of results from two independent experiments. MW, molecular weight.

In untreated C. glabrata cells, the CgRpn4_GFP fusion protein is distributed throughout the whole cell, with some level of nuclear signal (Fig. 4A). After fluconazole stress, an enrichment of nuclear localization was observed (Fig. 4B and C). Fluconazole treatment therefore changes the relative distribution of CgRpn4 to the nucleus. The activation of its target genes could be partially dependent on the translocation of the transcription factor. In S. cerevisiae, Rpn4 protein levels are regulated by the proteasome in a negative-feedback loop. Therefore, a decrease in the CgRpn4_GFP signal could occur due to the activation of proteasome genes by the transcription factor. However, our localization data appear to show steady levels of CgRpn4 production. To evaluate if the levels of CgRpn4 are being affected by a negative-feedback loop, Western blotting was performed before and after fluconazole treatment (Fig. 4D). The CgRpn4_GFP fusion protein could be detected at similar levels under both experimental conditions, which is consistent with the localization data. This shows that CgRpn4 plasmid-driven production contributes to the protein steady state.

### *CgRPN4* plays a role in the maintenance of ergosterol levels, membrane permeability, and fluconazole accumulation.

Transcriptomics analysis of the KUE100 wild-type C. glabrata strain during fluconazole stress revealed the significant activation of the ergosterol biosynthesis pathway mediated by CgRpn4. This led us to hypothesize that CgRpn4 may contribute to preserving ergosterol levels in C. glabrata upon fluconazole exposure.

C. glabrata cells were grown to the mid-exponential phase and transferred to fresh medium (control) or fresh medium containing 150 mg/liter fluconazole. After 4 h and 12 h of incubation, cells were collected, total ergosterol was extracted, and its levels were quantified by high-performance liquid chromatography (HPLC).

**FIG 5:**
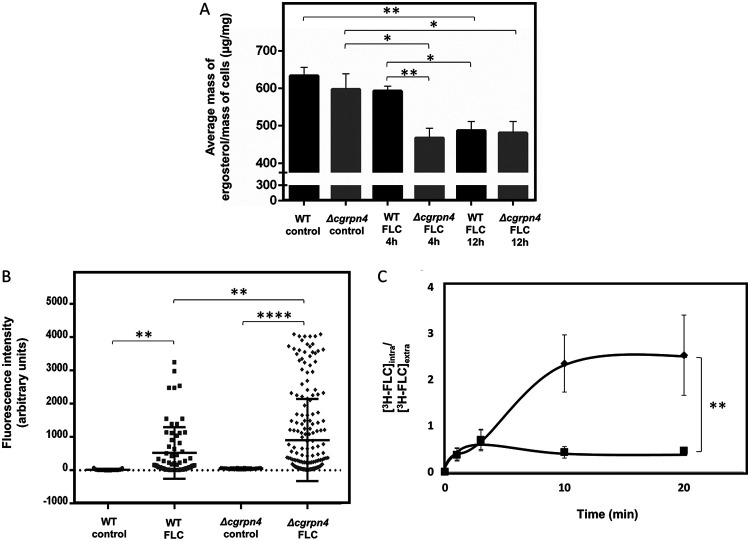
CgRpn4 regulates ergosterol levels in C. glabrata cells during fluconazole stress, affecting permeability and drug accumulation. (A) KUE100 and KUE100_Δ*cgrpn4* C. glabrata cells were harvested after 15 h of growth in RPMI 1640 medium (control) or after 4 h or 12 h of fluconazole stress. Ergosterol was extracted and quantified by HPLC. Cholesterol was used as an internal standard to evaluate the yield of ergosterol extraction. The displayed ergosterol contents are representative of results from at least six independent experiments. Error bars represent the corresponding standard deviations. *, *P* < 0.05; **, *P* < 0.01. (B) Comparison of plasma membrane permeabilities of exponential-phase KUE100 (squares) and KUE100_Δ*cgrpn4* (diamonds) cells under control conditions or upon 1 h of fluconazole stress. The estimation of plasma membrane permeability is based on the fluorescence intensity values exhibited by yeast cells upon the passive accumulation of propidium iodide. Error bars represent the corresponding standard deviations. **, *P* < 0.01; ***, *P* < 0.001. (C) Time course accumulation of radiolabeled [^3^H]fluconazole in strains KUE100 (squares) and KUE100_Δ*cgrpn4* (diamonds) during cultivation in liquid BM in the presence of 150 mg/liter unlabeled fluconazole. Accumulation values are the averages from at least three independent experiments. Error bars represent the corresponding standard deviations. **, *P* < 0.01.

Deleting *CgRPN4* does not affect ergosterol levels in untreated cells (Fig. 5A). The absence of *CgRPN4* reduces ergosterol levels upon fluconazole stress for 4 h or 12 h. Ergosterol levels in the wild type decreased only after 12 h and to levels comparable to those of the deletion strain, which indicates a relevant role of the transcription factor in this mechanism. Altogether, these data implicate CgRpn4 in the maintenance of the ergosterol content upon early fluconazole exposure. Moreover, these results show that CgRpn4, through transcriptional regulation of the ergosterol biosynthesis pathway, has a measurable effect on ergosterol levels during fluconazole stress, which contributes to antifungal resistance.

Cell permeability in response to fluconazole was investigated using the fluorescent probe propidium iodide (PI), and the possible participation of CgRpn4 was evaluated. Upon 1 h of exposure to fluconazole, C. glabrata cell permeability increases significantly (Fig. 5B). This observation is probably related to the inhibition of Erg11 by fluconazole, described previously to lead to the accumulation of the toxic sterol DMCDD that permeabilizes the plasma membrane (51, 52). The permeability of untreated Δ*cgrpn4* cells is not significantly different from that of wild-type cells. However, the permeability of Δ*cgrpn4* cells is significantly higher than that of the wild-type strain following fluconazole stress (Fig. 5B). These data indicate that fluconazole increases C. glabrata cell permeability and that CgRpn4 contributes to controlling its maintenance during fluconazole exposure, presumably by the upregulation of ergosterol biosynthesis.

The role of *CgRPN4* in ergosterol biosynthesis and plasma membrane permeability led us to investigate if this could be related to the increased azole susceptibility observed in the Δ*cgrpn4* mutant. Consistent with the observed susceptibility and cell permeability phenotypes, the Δ*cgrpn4* deletion mutant was found to accumulate 2.5-fold more radiolabeled fluconazole than the wild-type strain after 20 min of exposure to the antifungal (Fig. 5C). These results suggest that CgRpn4 activity mediates C. glabrata resistance to fluconazole by reducing its intracellular accumulation in yeast cells, possibly as a result of its regulation of ergosterol levels and membrane permeability.

### Determination of promoter recognition motifs by CgRpn4 and direct regulation of *CgERG11* expression.

**FIG 6:**
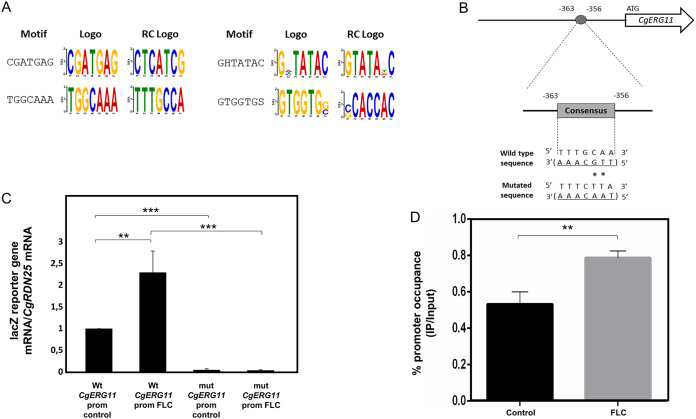
Possible CgRpn4 recognition sequence and direct regulation of *CgERG11*. (A) Motifs found to be overrepresented in the promoters of CgRpn4-activated genes, as found by the DREME tool. Sequences located upstream (bp −1000 to −1) of the genes found to be activated by CgRpn4 were retrieved from PathoYeastract (75) and submitted to DREME. The prediction was made using default parameters. The sequences shown correspond to the motifs outputted by DREME, excluding TATA box motifs. (B) The putative CgRpn4 recognition sequence in the promoter of *CgERG11* is in the complementary strand. The numbers refer to the position of the consensus site relative to the first ATG of the coding region. The wild-type sequence is shown underlined below the box, with asterisks denoting the base substitutions generated by site-directed mutagenesis. The resulting mutated sequence is shown below. (C) Comparison of the variations of *lacZ* transcript levels determined by RT-PCR in L5U1 cells harboring the pYEP354_Cg*ERG11*prom_*lacZ* or pYEP354_mut_Cg*ERG11*prom_*lacZ* plasmid under control conditions or after 1 h of fluconazole exposure. Transcript levels of *CgRDN25* were used for normalization. Expression values are the averages from at least three independent experiments. Error bars represent the corresponding standard deviations. **, *P* < 0.01; ***, *P* < 0.001. (D) ChIP–RT-PCR measurements of CgRpn4 promoter occupancy at the *CgERG11* promoter element containing the possible recognition motif. ChIP experiments were performed using mouse anti-c-Myc antibody and cultures of L5U1 cells harboring the pGREG526_PDC1_*CgRPN4* plasmid under control conditions or after 1 h of fluconazole exposure. The amplified amount of DNA was measured by RT-PCR and normalized to the total amount of the sample. Samples from the CgRpn4 IP were compared to the input (IP/input ratio) under each condition. To determine percent occupancy under each condition, values were calculated as the ratio of the percent precipitated under fluconazole stress to the percent precipitated under control conditions. Promoter occupancy values are the averages from three independent experiments. Error bars represent the corresponding standard deviations. **, *P* < 0.01.

In order to identify possible CgRpn4 DNA recognition sites, the promoters of CgRpn4-activated genes during fluconazole stress were searched for enriched motifs using DREME (53). Excluding TATA box sequences, 4 overrepresented motifs were found (Fig. 6A). Interestingly, one of the identified motifs (TGGCAAA) is identical or nearly identical to a core region of the ScRpn4 and CaRpn4 consensus (GGTGGCAAA and GAAGGCAAAA, respectively) found in promoters of proteasome genes (28, 50). This indicates that there is a high level of conservation in the promoter sequences recognized by Rpn4 across these species.

We found a nearly identical motif (TTGCAAA) located at positions −363 to −356 (Fig. 6B) of *CgERG11*. To determine if this motif is required for the activation of *CgERG11*, the *CgERG11* promoter was placed upstream of the *lacZ* reporter gene, and site-directed mutagenesis was used to disrupt the putative binding site.

Upon 1 h of fluconazole exposure, *lacZ* expression driven by the *CgERG11* promoter increased 2-fold in comparison to control conditions (Fig. 6C), which is in accordance with our transcriptomics data. When the TTGCAAA motif in the *CgERG11* promoter was disrupted by 2 nucleotide substitutions (from TTGCAAA to TAACAAA), *lacZ* expression was reduced by 20-fold compared to the wild-type promoter under control conditions and even more so after fluconazole exposure (59-fold) (Fig. 6C). These results indicate that the identified motif is required for *CgERG11* basal expression and especially *CgERG11* activation during fluconazole stress. Chromatin immunoprecipitation (ChIP) followed by RT-PCR was used to establish a direct link between CgRpn4 and the identified *CgERG11* promoter motif using a CgRpn4_c-Myc fusion protein expressed via the pGREG526_PDC1_*CgRPN4* plasmid. The results show CgRpn4 can bind the *CgERG11* promoter in the region containing the TTGCAAA motif (Fig. 6D). The promoter is bound by CgRpn4 under control conditions and increases its promoter occupancy during fluconazole stress, strongly suggesting direct CgRpn4 binding to this essential motif in the *CgERG11* promoter.

## DISCUSSION

C. glabrata is an emerging fungal pathogen with an impressive ability to acquire resistance to azole antifungal drugs. An understanding of the molecular basis of this phenotype is crucial for designing better-suited therapeutic approaches to tackle infections caused by C. glabrata.

In this study, we present a novel pathway used by C. glabrata to respond and adapt to azole drugs under the control of the transcription factor CgRpn4. Deletion of *CgRPN4* increases C. glabrata susceptibility to multiple azole drugs. According to the latest EUCAST breakpoints, the attained MIC values represent decreased susceptibility to fluconazole, although not a shift from resistant to intermediate, as the parental strain used exhibits an intermediate fluconazole susceptibility profile. In the closely related yeast S. cerevisiae and in the pathogenic species C. albicans, Rpn4 is a regulator of proteasome genes (27, 28, 50). Defects in proteasome levels can potentially impact a wide range of phenotypes and play a role in the general stress response. To test this possibility, the susceptibility of the Δ*cgrpn4* deletion mutant to additional antifungal drugs and other stress agents was assessed. Notably, the results indicate that CgRpn4 functions mainly as a regulator of azole resistance.

RNA sequencing (RNA-seq)-based transcriptomics revealed that Rpn4 is also a regulator of proteasome genes in C. glabrata, thus unveiling a highly conserved physiological function among yeast species. Additionally, the activation of *ERG* genes, including *CgERG11*, was identified as a key underlying mechanism of CgRpn4-mediated fluconazole resistance. These findings are supported by our data as well as data from other studies showing *ERG* gene upregulation during azole stress (42–47). Moreover, the upregulation of *CgERG11* was found to be dependent on a promoter motif recognized and bound by CgRpn4. Together with the transcriptomics data, the essential role of the TTGCAAA motif in *CgERG11* activation and the direct binding of CgRpn4 (especially under fluconazole exposure) to the *CgERG11* promoter element provide evidence for a prevalent regulatory role in ergosterol biosynthesis regulation leading to fluconazole resistance.

The activation of the ergosterol biosynthesis pathways was seen to have a measurable impact on the ergosterol levels of C. glabrata during fluconazole exposure. The deletion of *CgRPN4* leads to a reduction of ergosterol levels in the presence of fluconazole, indicating that the activation of ergosterol biosynthesis genes during fluconazole stress may be part of a compensatory mechanism to counteract ergosterol synthesis targeted by the antifungal. A similar mechanism has been reported for C. albicans, where fluconazole-tolerant strains present higher levels of intermediate metabolites of the sterol biosynthetic pathway and ergosterol ester, which was associated with the upregulation of ergosterol biosynthetic pathway genes (54). Furthermore, a reduction in the content of sphingolipids or ergosterol results in enhanced susceptibility to drugs (55–57). Consistent with this hypothesis, C. glabrata cells are more permeable and accumulate more intracellular fluconazole when *CgRPN4* is deleted, which is in good agreement with the role of this TF in the activation of ergosterol biosynthesis. In S. cerevisiae, *ScRPN4* is transcriptionally regulated under various stresses, and ScRpn4 protein levels are also controlled by proteasome activity in a negative-feedback loop (27). In our study, constant protein steady-state levels of CgRpn4 were detected, although it is possible that the efficient expression of the CgRpn4 protein by our expression system hindered the ability to detect turnover changes. As these data relate to the protein stability of CgRpn4, a possible feedback loop at the transcriptional regulation of *CgRPN4* remains to be established.

Here, we show the role of the transcription factor CgRpn4 in mediating fluconazole resistance in C. glabrata. Through transcriptional control over the ergosterol biosynthesis pathway, CgRpn4 regulates ergosterol levels in the plasma membrane, thus affecting cell permeability and fluconazole accumulation. Additionally, CgRpn4 exerts direct control over *CgERG11* expression, reinforcing its role as a relevant player in C. glabrata fluconazole resistance, which is likely conserved among other yeast species. Based on these results, we propose that CgRpn4 is a promising target to tackle the acquisition of azole drug resistance.

## MATERIALS AND METHODS

### Strains, plasmids, and growth media.

Candida glabrata parental strains KUE100 (58) and L5U1 (*cgura3*Δ*0 cgleu2*Δ*0*) (59) and derived single-deletion mutants were batch cultured at 30°C at 250 rpm in basal medium (BM) (20 g/liter glucose, 2.7 g/liter ammonium sulfate, and 1.7 g/liter yeast nitrogen base [YNB] without ammonia or amino acids) or RPMI 1640–2% glucose medium at pH 7.0. For BM assays using L5U1 transformed with either the pGREG576 or pGREG526 plasmid (harboring a URA3 selection marker), the medium was supplemented with 60 mg/liter leucine. The plasmids pGREG576 and pGREG526 were obtained from the Drag&Drop collection (60). The Saccharomyces cerevisiae BY4741 strain (MAT**a** *his3*Δ*1 leu2*Δ*0 met15*Δ*0 ura3*Δ*0*) was batch cultured at 30°C at 250 rpm in yeast extract-peptone-dextrose (YPD) medium (20 g/liter glucose, 20 g/liter peptone, 10 g/liter yeast extract).

### Disruption of *CgRPN4*.

The deletion of C. glabrata *RPN4* addressed in this study was carried out in the parental strain KUE100 using the method described previously by Ueno et al. (61). The primers used are presented in Table S4 in the supplemental material.

### Cloning of the C. glabrata *CgRPN4* gene (ORF *CAGL0K01727g*).

The pGREG576 and pGREG526 plasmids from the Drag&Drop collection were used as described previously to clone and express the C. glabrata open reading frame (ORF) *CAGL0K01727g* (18, 22, 62–65), giving rise to pGREG576_*CgRPN4* or pGREG526_*CgRPN4*. The *GAL1* promoter present in each plasmid was replaced by the copper-inducible *MTI* C. glabrata promoter (pGREG576_*CgRPN4*) or the constitutive *PDC1* C. glabrata promoter (pGREG526_*CgRPN4*) (66), giving rise to the pGREG576_MTI_*CgRPN4* or pGREG526_PDC1_*CgRPN4* plasmid. The primers used are presented in Table S4. The recombinant plasmids were obtained through homologous recombination in S. cerevisiae and verified by DNA sequencing.

### Antifungal susceptibility assays.

The susceptibility of the parental strain KUE100 to inhibitory concentrations of the selected drugs was compared to that of the deletion mutant KUE100_Δ*cgrpn4* by spot assays. The ability of *CgRPN4* gene expression to increase wild-type resistance to the tested chemical stresses was also examined in the URA3^−^ L5U1 C. glabrata strain using the pGREG576_MTI_*CgRPN4* centromeric plasmid.

Cell suspension preparation and spot assays were carried as described previously (18, 22, 62–65). The tested drugs included the following compounds, used in the specified concentration ranges that were found to exert inhibitory growth effects: the azole antifungal drugs ketoconazole (10 to 60 mg/liter), fluconazole (100 to 250 mg/liter), miconazole (0.10 to 0.50 mg/liter), itraconazole (15 to 30 mg/liter), clotrimazole (2.5 to 15 mg/liter), and tioconazole (0.30 to 0.70 mg/liter) (all from Sigma). Antifungals from other families included the polyene amphotericin B (0.05 to 0.20 mg/liter), the pyrimidine analog 5-FC (0.10 to 0.30 mg/liter), and the broad-activity fungicide mancozeb (0.5 to 1.5 mg/liter) (all from Sigma). The following osmotic and oxidative stresses were tested at levels found to exert inhibitory growth effects: NaCl (0.5 to 2 M) (from Panreac) and H_2_O_2_ (7.5 to 15 mM) (from Sigma), respectively. The MIC values of each antifungal were determined according to the EUCAST susceptibility testing method (67). Drugs not present in the reference method (e.g., ketoconazole) were used according to the same experimental procedures. The MIC assays were performed in 96-well plates containing RPMI 1640–2% glucose medium with the appropriate drug concentrations. Cells grown for 18 h were used to create an initial inoculum containing 5 × 10^6^ CFU/ml in distilled water, which was subsequently used to prepare a 5 × 10^5^-CFU/ml working suspension. The plates were then inoculated with the cell suspension to a final inoculum density of 2.5 × 10^5^ CFU/ml and incubated at 37°C for 24 h. Final cell growth was assessed by absorbance measurement at a 530-nm wavelength.

### CgRpn4 subcellular localization assessment.

The subcellular localization of the CgRpn4 protein was determined based on the observation of L5U1 C. glabrata cells transformed with the pGREG576_MTI_*CgRPN4* plasmid. These cells express the CgRpn4_GFP fusion protein, whose localization may be determined using fluorescence microscopy as described previously (18, 22, 62–65). C. glabrata cell suspensions were prepared in BM supplemented with leucine and 50 μM CuSO_4_, until a standard culture optical density at 600 nm (OD_600_) of 0.5 was reached, and transferred to the same medium with or without fluconazole. After 1 h of incubation, 1 drop of NucRed Live 647 was added to 1 ml of 4 × 10^7^ cells/ml, and cell suspensions were incubated in the dark with orbital agitation (30 min at 250 rpm). Cells were centrifuged (17,500 × *g* for 5 min), washed twice, and resuspended in phosphate-buffered saline (PBS) for final aliquots of 10^7^ cells/ml. The distribution of the CgRpn4_GFP fusion protein in yeast cells was determined by fluorescence microscopy with a Zeiss Axioplan microscope (Carl Zeiss MicroImaging) using excitation and emission wavelengths of 395 and 509 nm (GFP) or 541 and 686 nm (NucRed). Fluorescence images were captured using a cooled Zeiss AxioCam 503 color camera (Carl Zeiss Microscopy). For the determination of nuclear signal intensity, fluorescence intensity was defined as the average pixel-by-pixel intensity in the selected region of interest (nucleus) after the deduction of the cytoplasm pixel intensity. A minimum of 100 cells per experiment were used. The fluorescence images were background corrected by using dark-current images.

### Total RNA extraction.

C. glabrata strains KUE100 and KUE100_Δ*cgrpn4* were grown in BM until mid-exponential phase. Subsequently, cells were transferred to fresh medium (control) or fresh medium containing 150 mg/liter fluconazole and harvested after 1 h of incubation. Total RNA was isolated using an Ambion RiboPure yeast RNA kit according to the manufacturer’s instructions.

### Library preparation and gene expression analysis.

Strand-specific RNA-seq library preparation and sequencing were carried out as a paid service by the next-generation sequencing (NGS) core of the Oklahoma Medical Research Foundation, Oklahoma City, OK. Paired-end reads (Illumina HiSeq 3000 PE150, 2 by 150 bp, with 2 Gb of clean data) were obtained from KUE100 and KUE100_Δ*cgrpn4*. Two replicates of each sample were obtained from three independent RNA isolations, which were subsequently pooled. Sample reads were trimmed using Skewer (v0.2.2) (68) and aligned to the C. glabrata CBS138 reference genome, obtained from the Candida Genome Database (CGD) (http://www.candidagenome.org/), using TopHat (v2.1.1) (69) with the parameters –p 12 (number of threads), –g 1 (maximum number of times that a read can be mapped to the genome), –b2-very-sensitive (preset option), and –library-type fr-firststrand (to account for strand specificity). HTSeq (v0.7.1) (70) was used to count mapped reads per ORF. Differentially expressed genes were identified using DESeq2 (71), with an adjusted *P* value threshold of 0.05 and log_2_-fold change thresholds of −0.5 and 0.5. Default parameters in DESeq2 were used. Candida albicans and Saccharomyces cerevisiae homologs were obtained from the Candida Genome Database and Saccharomyces Genome Database (SGD) (https://www.yeastgenome.org/), respectively.

### Ergosterol quantification.

Ergosterol was extracted from cells using methods adapted from the ones described previously by Gong et al. (72) and carried out as described previously (73). Cells were cultivated in RPMI 1640–2% glucose medium with orbital agitation (250 rpm) until a standard culture OD_600_ of 5.0 was reached, harvested by centrifugation, and resuspended in 5 ml of methanol. One milliliter of a solution of 1 mg/ml of cholesterol (Sigma) was added as an internal standard to estimate the ergosterol extraction yield. Homogenization was carried out with glass beads for 30 s, followed by incubation at 320 rpm for 1 h. Each sample was then centrifuged, and 1.7 ml of the supernatant was collected, clarified, and stored until HPLC analysis. The extracts were separated in a 250-mm by 4-mm C_18_ column (LiChroCART Purospher Star RP-18 end-capped 5-μm column) at 30°C. Samples were eluted in 100% methanol at a flow rate of 1 ml/min. The detection of cholesterol and ergosterol was performed using a UV-visible (UV-Vis) detector set at 282 and 210 nm, respectively. Under the conditions used, the retention time of cholesterol was 15.4 ± 0.4 min, while ergosterol was eluted at 12.5 ± 0.2 min. Subsequent quantification of the two lipids was performed using appropriate calibration curves. The results are shown as micrograms of ergosterol per milligram of wet cell weight.

### Plasma membrane permeability.

Plasma membrane permeability was assessed by the passive uptake of propidium iodide (PI) (20 mM in dimethyl sulfoxide [DMSO]; Invitrogen). C. glabrata cell suspensions from strains KUE100 and KUE100_Δ*cgrpn4* were prepared in BM until a standard culture OD_600_ of 0.5 was reached and transferred to the same medium with or without 150 mg/liter fluconazole. After 1 h of incubation, PI was added to 1 ml of 4 × 10^7^ cells/ml to a final concentration of 20 μM, and cell suspensions were incubated in the dark with orbital agitation (15 min at 250 rpm). Cells exposed to PI were centrifuged (17,500 × *g* for 5 min), washed twice, and resuspended in PBS for final aliquots of 10^7^ cells/ml. PI fluorescence was detected by fluorescence microscopy with a Zeiss Axioplan microscope (Carl Zeiss MicroImaging), using excitation and emission wavelengths of 536 and 595 nm, respectively. Fluorescence images were captured using a cooled Zeiss AxioCam 503 color camera (Carl Zeiss Microscopy), and the images were analyzed with ZEN lite software from Zeiss Microscopy. The cell-to-cell fluorescence intensity was defined as the average pixel-by-pixel intensity in the selected region of interest, and a minimum of 100 cells per experiment were used. The fluorescence images were background corrected by using dark-current images.

### [^3^H]fluconazole accumulation assays.

[^3^H]fluconazole transport assays were carried out as described previously for other radiolabeled compounds (18, 22, 62–65). The internal accumulation of fluconazole was determined by calculating the ratio of the radiolabeled fluconazole measured within the yeast cells to that in the external medium (intracellular/extracellular). The parental strain KUE100 and the mutant strain KUE100_Δ*cgrpn4* were grown in BM until mid-exponential phase and harvested by filtration. Cells were washed and resuspended in BM to obtain dense cell suspensions (OD_600_ = 0.5, equivalent to approximately 1.57 mg [dry weight] ml^−1^). Readily, 0.1 μM [^3^H]fluconazole (1 mCi/ml) (Moravek Inc.) and 150 mg/liter of unlabeled fluconazole were added to the cell suspensions. Incubation proceeded for an additional 20 min. The intracellular accumulation of labeled fluconazole was monitored by filtering 200 μl of the cell suspension, at adequate time intervals, through prewetted glass microfiber filters (Whatman GF/C). The filters were washed with ice-cold TM buffer [0.1 M 2-(N-morpholino)ethanesulfonic acid (Sigma)/41 mM Tris (Sigma)], and radioactivity was measured in a Beckman LS 5000TD scintillation counter. Extracellular [^3^H]fluconazole was estimated by radioactivity assessment of 50 μl of the supernatant. Nonspecific [^3^H]fluconazole adsorption to the filters and to the cells (less than 5% of the total radioactivity) was assessed and taken into consideration. To calculate the intracellular concentration of labeled fluconazole, the internal cell volume (*V*_i_) of the exponential cells, grown in the absence of the drug and used for accumulation assays, was considered constant and equal to 2.5 mg (dry weight) μl^−1^ (74).

### *In silico* prediction of overrepresented sequences in CgRpn4-activated promoters.

The promoters (bp −1000 to −1) upstream of the coding regions of genes whose expression was found to be activated by CgRpn4 were retrieved using the Retrieve Upstream Sequence tool from PathoYeastract (75). The obtained sequences were submitted to DREME (MEME suite) (53) for the discovery of enriched sequences, using default parameters. The identified motifs were then cross-checked with ScRpn4 and CaRpn4 motifs retrieved from the TF-Consensus List tool from YEASTRACT (76, 77) and PathoYeastract (75), respectively.

### Cloning of the *CgERG11* promoter and site-directed mutagenesis.

The pYPE354 plasmid was used as described previously to clone and express the *lacZ* reporter gene (18). pYEP354 contains the yeast selectable marker *URA3* and the bacterial selectable marker *ampR. CgERG11* promoter DNA was generated by PCR using genomic DNA extracted from the sequenced CBS138 C. glabrata strain and primers presented in Table S4. The first primer contains a region with homology within the beginning of the *CgERG11* promoter and a recognition site for the EcoRI restriction enzyme, flanked by additional bases. The second primer contains a region with homology within the end of the *CgERG11* promoter and the beginning of the *CgERG11* coding sequence and a recognition site for the PstI restriction enzyme, flanked by additional bases. The amplified fragment was ligated into the pYEP354 vector (T4 ligase; New England BioLabs), previously cut with the same restriction enzymes, to obtain the pYEP354_*CgERG11*prom_*lacZ* plasmid. The putative CgRpn4 consensus sequence in the *CgERG11* promoter was mutated by site-directed mutagenesis using the primers listed in Table S4. The designed primers contain two mutations within the potential consensus sequence, resulting in the production of the mutated consensus sequence by PCR amplification to obtain the pYEP354_mut_*CgERG11*prom_*lacZ* plasmid. The original template was then degraded by DpnI digestion.

### RT-PCR gene expression measurement.

The transcript levels of the *CgERG11* or the *lacZ* reporter gene encoding β-galactosidase were determined by quantitative real-time PCR (RT-PCR). For *CgERG11* expression measurements, the KUE100 and KUE100_Δ*cgrpn4* strains were grown in BM until mid-exponential phase, transferred to fresh medium (control) or fresh medium containing 150 mg/liter fluconazole (OD600 = 0.5), and harvested after 1 h of incubation. Samples were immediately frozen at −80°C until RNA extraction. For *lacZ* expression measurements, L5U1 cells transformed with the pYEP354_*CgERG11*prom_*lacZ* or pYEP354_mut_*CgERG11*prom_*lacZ* plasmid were grown in BM supplemented with leucine until mid-exponential phase. Fluconazole exposure, cell harvesting, and storage were performed as mentioned above. For total RNA extraction, the hot-phenol method was applied (78). RT-PCR was performed as described previously (18, 22, 62–65). The synthesis of cDNA for RT-PCR experiments, from total RNA samples, was performed using the MultiScribe reverse transcriptase kit (Applied Biosystems) and the 7500 RT-PCR thermal cycler block (Applied Biosystems) according to the manufacturer’s instructions. The quantity of cDNA for the following reactions was kept at around 10 ng. The subsequent RT-PCR step was carried out using SYBR green (NZYTech) reagents with default parameters established by the manufacturer and the primers in Table S4. The *CgRDN25* gene transcript levels were used as an internal reference.

### Western blot analysis.

For Western blot analysis, L5U1 C. glabrata cells transformed with the pGREG576_MTI_*CgRPN4* plasmid were grown to mid-exponential phase in BM supplemented with leucine and 50 μM Cu_2_SO_4_ to induce fusion protein expression and then transferred to fresh medium (control) or fresh medium containing 150 mg/liter fluconazole. After 1 h of incubation, cells were collected and washed twice with cold PBS (pH 7.4). For protein extraction, a TCA (trichloroacetic acid) precipitation protocol was used. Briefly, cell pellets were resuspended in resuspension buffer (1.85 M NaOH, 7.5% 2-mercaptoethanol) by vortexing, followed by a 2-min incubation on ice (5 times). Proteins were precipitated with 25% TCA using 5 cycles of vortexing and 2 min of incubation on ice, followed by centrifugation. The pellets were washed with acetone and resuspended in Laemmli buffer at 65°C for 20 min (with rotation), followed by centrifugation, and the supernatant containing the protein extract was collected. Samples were separated on 12.5% sodium dodecyl sulfate-polyacrylamide gel electrophoresis (SDS-PAGE) gels. Proteins were transferred to an Amersham Protran 0.2-μm nitrocellulose membrane (GE Healthcare). Immunoblotting was performed using a 1:200 dilution of mouse IgG anti-GFP antibody (catalog number sc-9996; Santa Cruz Biotechnology Inc.) and a 1:1,000 dilution of anti-mouse IgG BP-HRP (binding protein conjugated to horseradish peroxidase) (catalog number sc-516102; Santa Cruz Biotechnology Inc.). Detection was performed using Fusion Solo (Vilber) following incubation with an enhanced chemiluminescence (ECL) substrate.

### CgRpn4 chromatin immunoprecipitation assays followed by RT-PCR.

L5U1 C. glabrata cells transformed with the pGREG526_PDC1_*CgRPN4* plasmid were grown to mid-exponential phase in BM supplemented with leucine until 100 OD units were reached and then transferred to fresh medium (control) or fresh medium containing 150 mg/liter fluconazole. After 1 h of incubation, cells were cross-linked with 1% formaldehyde (15 min), and cross-linking was quenched with 340 mM glycine (10 min). Cells were collected, washed twice with cold PBS (pH 7.4), and frozen immediately at −80°C. Cell pellets were lysed with 0.5-mm zirconia beads (Invitrogen) in lysis buffer (50 mM HEPES [pH 7.5], 140 mM NaCl, 1 mM EDTA, 1% Triton X-100, 0.5% NP-40, 0.5 mM dithiothreitol [DTT]) by vortexing for 20 s and incubation on ice for 2 min (4 times). Cell debris was pelleted by centrifugation, and the lysate was collected. Lysates were sonicated four times for 2 min followed by 2 min on ice using a Bioruptor water bath sonicator. Sonicated lysates were cleared by centrifugation, and 5% of the sample was collected for use as the input (sonicated chromatin, not immunoprecipitated). For immunoprecipitation (IP), chromatin was incubated overnight at 4°C (with rotation) with protein G SureBeads (Bio-Rad) coupled with IgG anti-c-Myc antibody (catalog number sc-40; Santa Cruz Biotechnology Inc.). Beads were washed according to the manufacturer’s instructions, and the samples were eluted at 65°C for 20 min in Tris-EDTA (TE) buffer with 0.5% SDS. Input and immunoprecipitated samples were incubated overnight at 65°C for cross-linking reversal. DNA was purified with the NZYGelPure kit (NZYTech). Quantification of a specific *CgERG11* promoter region was performed by RT-PCR using 5 μl of the DNA preparation from each reaction mixture. The primers used are listed in Table S4. Immunoprecipitated samples were processed together with the input samples, and the amplification product was normalized to the total template used. Based on methods reported previously (79, 80), the ratio of the immunoprecipitated promoter to the input (IP/input) was calculated, and ratios in fluconazole samples were compared to the ratio in the control samples at the specific locus of the *CgERG11* promoter.

### Statistical analyses.

All experiments represent the averages from two or more independent experiments, with at least two technical replicates each. Error bars represent standard deviations. Statistical analyses were performed using one-way analysis of variance (ANOVA) with Tukey’s correction (comparisons of more than 2 groups) or two-tailed *t* tests with Welch’s correction (comparisons of 2 groups). Significance levels are indicated in the figure legends (*, *P* < 0.05; **, *P* < 0.01; ***, *P* < 0.001; ****, *P* < 0.0001).

### Data availability.

Raw data are available at the Gene Expression Omnibus (GEO) under accession number GSE129349.

## Supplementary Material

Supplemental file 1

Supplemental file 2

Supplemental file 3

Supplemental file 4
