# Supplementary material for: Candida glabrata Transcription Factor Rpn4 Mediates Fluconazole Resistance through Regulation of Ergosterol Biosynthesis and Plasma Membrane Permeability
Source: Antimicrob Agents Chemother. 2020 Aug 20;64(9):e00554-20. doi: 10.1128/AAC.00554-20 (PMC7449212; doi:10.1128/AAC.00554-20)
Supplement: Supplemental file 4 [file AAC.00554-20-s0004.pdf]

SUPPLEMENTAL MATERIAL

Supplemental Figure S1 Growth experiments with relevant cell and fluconazole concentrations

Supplemental Table S1 List of differentially expressed genes in WT FLC/WT control samples

Supplemental Table S2 List of differentially expressed genes in *Δcgrpn4* FLC/WT FLC samples

Supplemental Table S3 List of differentially expressed genes in *Δcgrpn4* control/WT control samples

Supplemental Table S4 List of primers used in this study

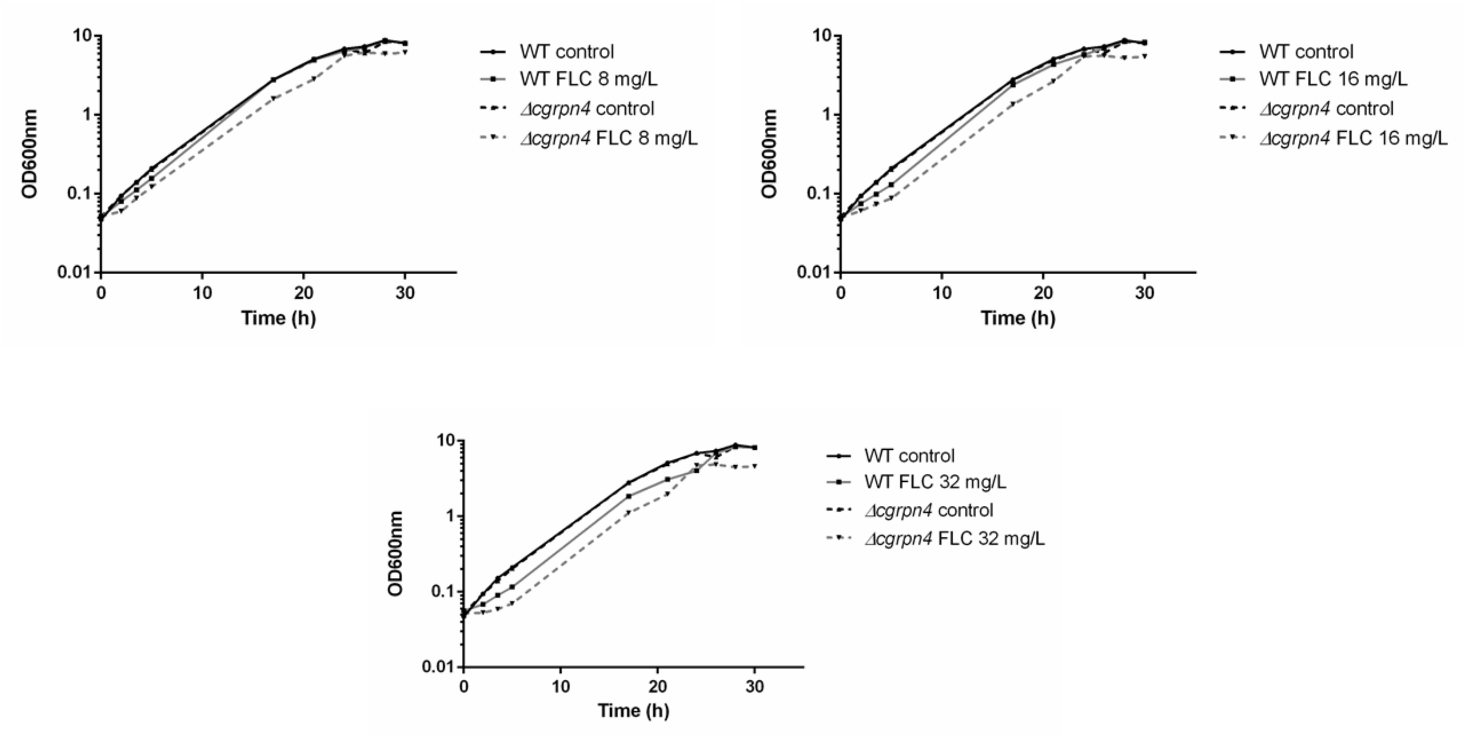

Figure S1 – *CgRPN4* is required for adaptation to fluconazole stress in *C. glabrata*. Comparison of the susceptibility of KUE100 and KUE100_*Δcgrpn4* strains during cultivation in liquid BM in the presence of varying concentrations of fluconazole. Cells were grown to mid-exponential phase in BM and then transferred to fresh medium (control) or to fresh medium containing 8, 16 or 32 mg/L fluconazole ($OD600_{nm}$ = 0.05). Growth curves are representative of at least 2 independent experiments.

Supplemental Table S4 List of primers used in this study

Primer Name/Function	Primer sequence
CgHIS3 replacement cassette	Fw - 5'-TATATAAAGGAGCACTGGTGGCGGTGGTGGCAATTCTATTAAAACTTTCCTCTCGAGGGCCGCTGATCACG-3'<br>Rv - 5'-AAAAAAAAATTATAATGATGTATCCGAAATTTTAAAAGAAATTTGAATGATGTTGGCATCGTGAGGCTGG-3'
CgRPN4 deletion confirmation	Fw - 5'-GATTTGGGACTGAAGAGG-3'<br>Rv - 5'-ATAGTCCACATCGTCGTAG-3'<br>Check - 5'-TCCCTTTCTGATGTGGTGGC-3'
Cloning of *CgRPN4* in pGREG526/576	Fw - 5'-GAATTCGATATCAAGCTTATCGATACCGTCGACAATGACGTCTATAGATTTGGGAC-3'<br>Rv - 5-GCGTGACATAACTAATTACATGACTCGAGGTCGACTTATGCAGTGACAAATCCGATG-3'
Cloning of *MTI* promoter in pGREG576	Fw - 5'-TTAACCCTCACTAAAGGGAACAAAAGCTGGAGCTCCTGTACGACACGCATCATGTGGCAATC-3'<br>Rv - 5'-GAAAAGTTCTTCTCCTTTACTCATACTAGTGCGGCTGTGTTTGTTTTTGTATGTGTTTGTTG-3'
Cloning of *PDC1* promoter in pGREG526	Fw - 5'-TTAACCCTCACTAAAGGGAACAAAAGCTGGAGCTAGCATTTTTATACACGTTTTAC-3'<br>Rv - 5-ACCGTTAATTAACCCGGGGATCATACTAGTGCGGCTGTTAATGTTTTTTGGCAATTG-3'
Cloning of *CgERG11* promoter in pYEP354	Fw - 5'-CCGGAATTCGGTCGACTACTTCAAGGCTA-3'<br>Rv - 5'-AACTGCAGCTTCAGTGGACATGTTATTGT-3'
CgERG11 promoter mutagenesis in pYEP354	Fw - 5'-GAGTGACATCTTTGTTAGCCCTTCCCATC-3'<br>Rv - 5'-GATGGGAAGGGCTAACAAAGATGTCACTC-3'
CgERG11 gene expression measurement	Fw - 5'-GCCATTGGCGCAGAGAGT-3'<br>Rv - 5'-GCCATGTGATGGTGTACACAAAT-3'
lacZ gene expression measurement	Fw - 5'-TGGCTGGAGTGCGATCTTC-3'<br>Rv - 5'-CGTGCATCTGCCAGTTTGAC-3'
CgRDN25 gene expression measurement	Fw - 5'-AACAACTCACCGGCCGAAT-3'<br>Rv - 5'-CAAGCGTGTTACCTATACTCCGCCGTCA-3'
CgERG11 promoter amplification after ChIP	Fw - 5'-CTGCACCAAACGACATGAGA-3'<br>Rv - 5'-GTGCTGAGCAAAGGCCATAA-3'
